# Polarization nano-tomography of tightly focused light landscapes by self-assembled monolayers

**DOI:** 10.1038/s41467-019-12127-3

**Published:** 2019-09-20

**Authors:** Eileen Otte, Kemal Tekce, Sebastian Lamping, Bart Jan Ravoo, Cornelia Denz

**Affiliations:** 1 Institute of Applied Physics, University of Muenster, Corrensstr. 2/4, 48149 Muenster, Germany; 2 Organic Chemistry Institute and Center for Soft Nanoscience, University of Muenster, Corrensstr. 40, 48149 Muenster, Germany

**Keywords:** Applied optics, Optical materials and structures, Other photonics

## Abstract

Recently, four-dimensional (4D) functional nano-materials have attracted considerable attention due to their impact in cutting-edge fields such as nano-(opto)electronics, -biotechnology or -biomedicine. Prominent optical functionalizations, representing the fourth dimension, require precisely tailored light fields for its optimal implementation. These fields need to be like-wise 4D, i.e., nano-structured in three-dimensional (3D) space while polarization embeds additional longitudinal components. Though a couple of approaches to realize 4D fields have been suggested, their breakthrough is impeded by a lack of appropriate analysis techniques. Combining molecular self-assembly, i.e., nano-chemistry, and nano-optics, we propose a polarization nano-tomography of respective fields using the functional material itself as a sensor. Our method allows a single-shot identification of non-paraxial light fields at nano-scale resolution without any data post-processing. We prove its functionality numerically and experimentally, elucidating its amplitude, phase and 3D polarization sensitivity. We analyze non-paraxial field properties, demonstrating our method’s capability and potential for next generation 4D materials.

## Abstract (web summary)

The realisation of 4D light fields, where longitudinal polarisation represents the fourth dimension, has been limited by the lack of appropriate analysis techniques. Here, the authors use interaction with self-assembled monolayers of fluorescent molecules, which allow for identification of non-paraxial light fields based on a single image frame.

## Introduction

Within the last decades, functionalized nano-systems have exceled contributing to future four-dimensional (4D) nano-materials and their applications in nano-(opto)electronics, -biotechnology, or -biomedicine^1–6^. For instance, peptides have been employed as functional components in nano-systems for disease treatments, or stimuli-responsive nano-carriers were applied for drug delivery^4,7^. Nowadays one can summarize the class of 4D materials generally as three-dimensionally (3D) nano-structured materials embedding an addressable functionality as fourth dimension. Most man-made nano-technology is based on top-down approaches governed by the concept of continuous miniaturization to the nano-scale. In contrast, nature implements a bottom-up approach as the prime strategy to construct dynamic, adaptive and learning systems at the nano-scale. This strategy includes self-assembly as an attractive route to the customization of 3D nano-structures that at the same time exhibit an electronic, magnetic, or optical functionality as fourth dimension^5,6^.

Leading functionalities are addressed optically as, e.g., light-based material changes in azobenzenes on surfaces, which requires a light field of appropriate characteristics for its optimal implementation. Therefore, light needs to be precisely tailored in all three spatial dimensions as well as in all its degrees of freedom, namely amplitude, phase, and 3D polarization. Thus, for optimally addressing a 4D nano-material, the respective light fields need to be likewise nano-structured in 4D, only achievable in the non-paraxial regime. Converting paraxial to non-paraxial light by, e.g., tightly focusing (numerical aperture (NA) ≥ 0.7), initial radial electric field components are tilted and transformed into non-negligible longitudinal field contributions^8,9^ as fourth dimension. Hence, focal 4D light fields are shaped^10–16^, which include complex topological structures as optical Möbius strips or ribbons^17–20^. Note that 3D polarization states and topologies as Möbius strips may also be realized by, e.g., off-axis interference of structured beams^21^. However, the desired nano-scale structure is achieved only in a tightly focused field. The 3D polarization nature as well as associated sub-diffractive, thus, nano-scale complexity of non-paraxial 4D fields^22,23^ represent the required tool for the effective implementation of novel 4D functional nano-materials.

However, the realization and application of these light fields is actually obstructed by the lack of appropriate analysis techniques. The benefit of using the complex 4D nature and nano-scale structure of non-paraxial fields represent at the same time a major difficulty impeding the application of metrology tools precisely working in the paraxial regime. Hence, there is an urgent demand for fast, thus, single-shot nano-tomographic techniques allowing for the immediate identification of focal fields including their amplitude, phase and 3D polarization. “Single-shot” refers to the fact that only a single measurement step, e.g., one camera image, is required for the field identification. Until now, a few limited approaches have been proposed^24–26^, but are failing to satisfy the topical demand since they are based on slow scanning techniques with multiple shots in combination with the requirement of precise knowledge of the scanning probe characteristics and extensive reconstruction algorithms.

Here, we show a single-shot nano-tomographic approach that does not require any post-measurement data processing for the identification and investigation of 4D light fields. For this purpose, we combine nano-chemistry and nano-optics to analyze light fields by the functional 4D nano-material itself as sensor. We apply nature inspired bottom-up assembly of fluorescent sulforhodamine B molecules for the creation of a functional molecular nano-system, or, more precisely, self-organized functionalized nano-surfaces^27,28^. We develop these self-assembled monolayers (SAMs)^29–31^ by exploiting the process of *π*–*π*-stacking, resulting in a fluorescent nano-tomographic detector. Crucially, respective fluorescence, i.e., the material’s fourth dimension, is sensitive to the amplitude, phase and 3D polarization of the exciting non-paraxial light field, which we prove numerically as well as experimentally. Hence, the created tool enables the qualitative single-shot analysis by a single camera image of complete transverse planes of a focal 4D field with spatial resolution at the nanometer-scale in all three spatial dimensions. Our approach finally enables the demanded experimental study of 4D structured fields, and thereby unlocks their potential arising in combination with 4D functional nano-systems.

## Results

### Tailored non-paraxial 4D light fields

The appropriate demonstration of the nano-tomographic approach requires representative 4D light fields, fully-structured^32–34^ in all its degrees of freedom. Hence, these fields need to embed amplitude, phase as well as polarization structuring of well-defined shape which can be tailored on demand. Therefore, we chose to customize non-paraxial light fields by holographic phase and polarization modulation in the paraxial regime. More precisely, we realize higher-order vector field of pure linear polarization with additional phase vortices and tightly focus these by a high-NA microscope objective (MO). Thereby, complexly shaped 4D fields of three-dimensional polarization ${{\cal{E}}}({x,y,z}) = [{\cal{E}}_{x}({x,y,z}),{\cal{E}}_{y}({x,y,z}),{\cal{E}}_{z}({x,y,z})]^{T}$ are formed with non-neglectable longitudinal polarization component ${\cal{E}}_z$. Note that also other kinds of fully-structured light fields could be applied for the demonstration of our nano-tomographic method’s operating principle. In the following we reason our choice of light fields.

**Fig. 1:**
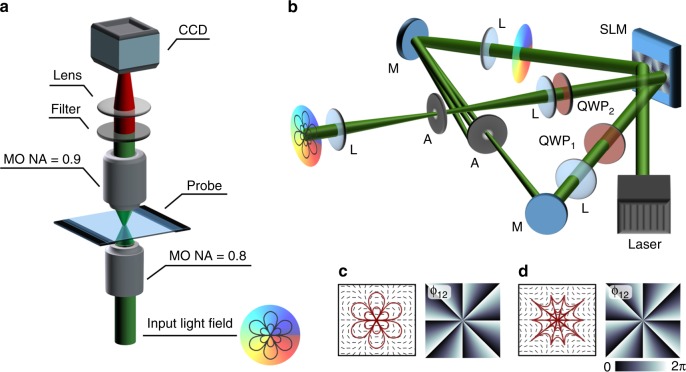
Concept of detecting non-paraxial light fields. **a** Microscope system for tightly focusing tailored paraxial light fields and detecting fluorescence; **b** setup for the generation of tailored vectorial light fields with additional phase vortices (paraxial regime; SLM: spatial light modulator, L: lens, M: mirror, A: aperture, QWP_1,2_: quarter wave plate, CCD camera); **c** vectorial flower and **d** spider web structure (*σ*_12_ = ±8), whose polarization distribution is indicated black lines (red: flow lines), with according phase Φ_12_ of complex Stokes field Σ_12_

**Fig. 2:**
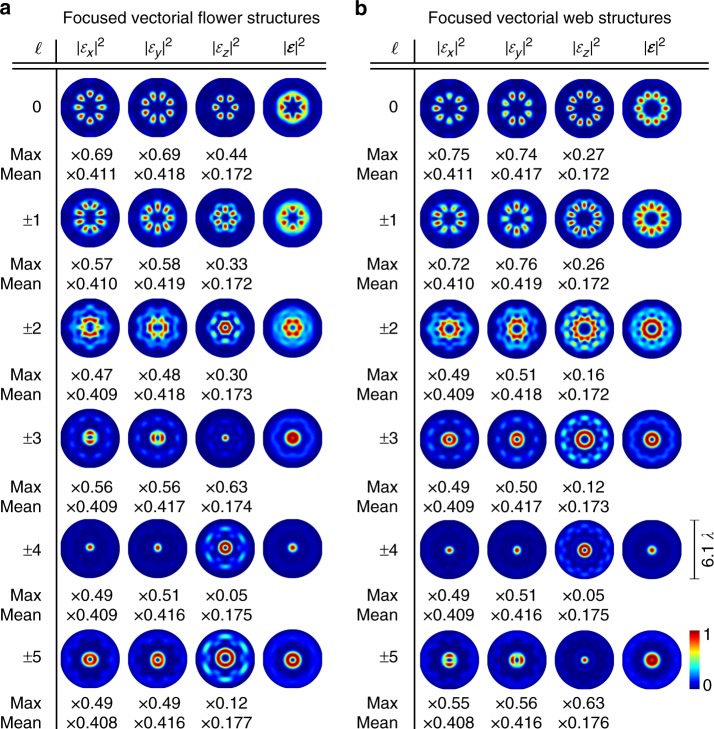
Numerics on tailored non-paraxial light fields ${{{\cal{E}}}}{({x},{y},{0})}$ realized by tightly focusing tailored vectorial fields. Vectorial flower (**a**) or web (**b**) structures (*σ*_12_ = ±8) with additional phase vortices of charge $\ell$ are considered as input fields. The total focal intensity $|{{{\cal{E}}}}|^2$ as well as intensity contributions $|{\cal{E}}_{x,y,z}|^2$ of transverse (${\cal{E}}_{x,y}$) and longitudinal (${\cal{E}}_z$) polarization components in the focal plane (*x*, *y*, 0) are presented (NA = 0.8). The relation of the maximum (max) or mean value of $|{\cal{E}}_{x,y,z}|^2$ to the maximum or mean of $|{{{\cal{E}}}}|^2$, respectively, is given below images

Higher-order vector fields are of point symmetric shape with its symmetry point representing an on-axis V-point singularity of undefined polarization^35–38^. This singularity or the respective field is characterized by the index *σ*_12_, defined according to the corresponding phase Φ_12_ of the complex Stokes field *Σ*_12_ = *S*_1_ + i*S*_2_ = *A*_12_ exp(iΦ_12_) (normalized Stokes vector **S** = [*S*_0_, *S*_1_, *S*_2_, *S*_3_]^*T*^) with $\sigma _{12} = {\oint}_c {\mathrm{d}} {\Phi} _{12}/2\pi\,(|\sigma_{12}|/2\in \mathbb{N})$^38–40^. Surrounding linear states of polarization form |*σ*_12_ − 2| flower petals (*σ*_12_ > 0) or spider web sectors (*σ*_12_ < 0), as exemplarily illustrated in Fig. 1c, d. As a crucial consequence, the ratio of azimuthal and radial components of flower- and web-shaped beams, being responsible for the occurrence of focal longitudinal electric field contributions, is dependent on the order *σ*_12_ of the singular light field, thus can be tailored precisely in amount and shape on demand^10^. As an illustrative example, well visualizing this property, we chose *σ*_12_ = ±8. Here, in the focal plane (*z* = 0), transverse and longitudinal focal electric field components feature conspicuous intensity configurations with |*σ*_12_| = 8 and |*σ*_12_ − 2| petals, respectively (Fig. 2). Further, the total focal intensity reveals a dark star (*σ*_12_ > 0) or bright flower (*σ*_12_ < 0) shape. Hence, shapes clearly differ for incident flower or web structures with sophisticated, recognizable structures in the non-paraxial regime, as demanded for the demonstration of our approach.

In order to highlight the SAM-based method’s sensitivity to amplitude, phase as well as polarization, we additionally include on-demand phase variation. For this purpose, we imprint additional global phase vortices of topological charge $\ell = \pm \{ 0,1,2,3,4,5\}$ onto the initial paraxial vector field. In Fig. 2, we present the respective numerical simulations (details on numerical approach can be found in the Methods) for the focused flower (a) and spider web (b) structure with additional vortices at *z* = 0. The total intensity $|{{{\cal{E}}}}|^2$ as well as the contributions per polarization component $|{\cal{E}}_{x,y,z}|^2$ in the focal plane are shown. All distributions are normalized by its own maximum, while the ratio of the maximum or mean value of $|{\cal{E}}_{x,y,z}|^2$ to the maximum or mean value of $|{{{\cal{E}}}}|^2$, respectively, is given below the images (max or mean). By the addition of phase vortices, we create a distinct difference in the relative phase of each focal electric field component (see next section) and clearly vary the shape as well as ratio of contributing components (for details see Methods). These differences are appropriate tools for testing the functionality of our nano-tomographic approach as they will cause characteristic fluorescence images for these field (see next section).

Different methods can be implemented for the experimental creation of fully-structured non-paraxial light fields^23^. For our system we chose a holographic approach because of the dynamic adaptability of created modes. We apply a holography-based dynamic modulation system^33,41^ in combination with a tightly focusing microscope configuration (for details see Methods), as visualized in Fig. 1a, b. By double passing a phase-only spatial light modulator (SLM) in split-screen-mode (Fig. 1b), we tailor the phase (first pass) as well as polarization (second pass) of light in the paraxial regime (wavelength *λ* = 532 nm). Note that the first half of the SLM is imaged on the second by a 4*f*-configuration. In the next step, the created beam is tightly focused by a high-NA MO (×100, NA = 0.8), so that a non-paraxial light field is formed. For this purpose, the second half of the SLM is imaged by 2*f*_1_–2*f*_2_ system on the back-aperture of the MO. By adapting the spatial phase or polarization information, encoded on the SLM, the realization as well as the dynamic customization of intended fully-structured 4D light fields is enabled.

### SAMs as nano-detectors

Up to now, non-paraxial 4D light fields as introduced above could not be analyzed as required for unlocking their potential in combination with 4D nano-materials. Solving this issue, here, we present the operating principle of our fast, single-shot method for the qualitative analysis of non-paraxial fields. For this polarization nano-tomography we apply a 4D material itself as nano-sensor, namely SAMs of fluorescent sulforhodamine B silane (maximal absorption: *λ*_abs_ = 572 nm; maximal emission: *λ*_fl_ = 594 nm) produced on a silica glass cover slip (for details see Methods and Supplementary Methods). Here, the property of fluorescence represents the fourth dimension embedded in the 3D nano-structured monolayer. Typically, a single molecule in a transverse (*x*, *y*)-plane reveals a fluorescence rate *R* according to^24^

1 $$R(x,{\kern 1pt} y) = a|{\mathbf{d}}\cdot {\mathrm{ }}{{{\cal{E}}}}(x,{\kern 1pt} y)|^2,$$

with the constant *a* depending on the absorption cross-section and quantum yield of the molecule. Further, **d** = [*d*_*x*_, *d*_*y*_, *d*_*z*_]^*T*^ represents the unit vector along the absorption dipole moment of the molecule. Considering a monolayer of random dipole orientation per fluorescent molecule, the vector **d** is replaced by transverse position dependent vector **d**(*x*, *y*). Note that *d*_*z*_ > 0 due to the molecules’ defined bonding site on the cover slip or, more precisely, silane (see Methods). This random dipole orientation on a micrometer level in combination with positive *d*_*z*_-value, achieved by self-assembly (see Methods), is the key feature of the SAM which enables its application as nano-sensor for non-paraxial fields, as it will turn out in the following.

One might expect to observe fluorescence resembling a disturbed image of the intensity $|{{{\cal{E}}}}|^2$ of the non-paraxial electric field in the chosen transverse plane, since the random dipole vector orientation may annihilate the polarization dependence of the fluorescence image. However, this is not the case, as, besides amplitude and polarization, one needs to consider the relative phases *φ*_*x*,*y*,*z*_ between contributing electric field components ${\cal{E}}_{x,y,z} = {\cal{E}}_{x,y,z}^0\exp ({\mathrm{i}}\varphi _{x,y,z})$. To emphasize this, we calculate the fluorescence rate assuming all dipoles to be oriented according to **d**(*x*, *y*) = *c*[1, 1, 1]^*T*^ (∀(*x*, *y*), normalization constant *c*), resulting in

2 $$R(x,{\kern 1pt} y) \propto |{\cal{E}}_x^\prime + {\cal{E}}_y^\prime + {\cal{E}}_z^\prime |^2.$$

Here, ${\cal{E}}_{x,y,z}^\prime$ inherit the amplitude and phase of ${\cal{E}}_{x,y,z}$, respectively, whereby ${\cal{E}}_{x,y,z}^\prime$ can be considered as scalar light fields of the same polarization emitted by the fluorescent dipoles. Hence, the resulting fluorescence will resemble the interference of the three scalar fields ${\cal{E}}_x^\prime$, ${\cal{E}}_y^\prime$, and ${\cal{E}}_z^\prime$.

**Fig. 3:**
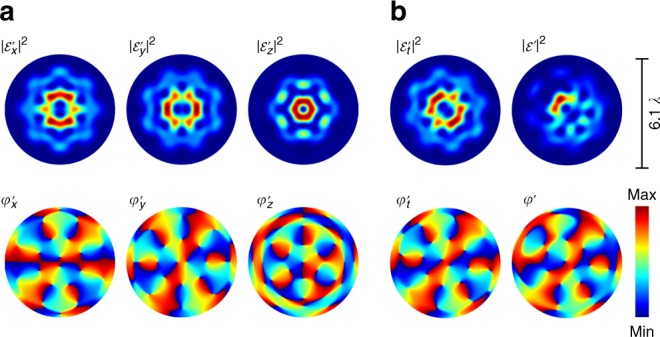
SAM fluorescence excited by non-paraxial light. Analysis for **d**(*x*, *y*) = *c*[1, 1, 1]^*T*^, ∀(*x*, *y*) and fluorescence caused by non-paraxial light fields with significant ${\cal{E}}_z$ contributions shown by the example of a focused flower structure (*σ*_12_ = 8) with additional phase vortex $\ell = 2$. **a** Normalized intensity $|{\cal{E}}_{x,y,z}^\prime |^2\:(\in [0,\,1])$ and relative phase $\varphi _{x,y,z}^\prime\:(\in[0,\,2\pi])$ contributions emitted by monolayer, excited by ${\cal{E}}_{x,y,z}$. **b** Normalized intensities $|{\cal{E}}_t^\prime |^2$ and $|{{{\cal{E}}}}^\prime |^2\:(\in [0,\,1])$ with relative phases $\varphi _t^\prime$ and $\varphi\prime (\in[0, \ 2\Pi])$ corresponding to ${\cal{E}}_t^\prime = {\cal{E}}_x^\prime + {\cal{E}}_y^\prime$ and ${{{\cal{E}}}}^\prime = {\cal{E}}_x^\prime + {\cal{E}}_y^\prime + {\cal{E}}_z^\prime$, respectively

As representative example, we apply a tightly focused vector beam with *σ*_12_ = 8 and $\ell = 2$ (Fig. 2a) as exciting light field at the focal plane (*z* = 0) with respective ${\cal{E}}_{x,y,z}^\prime$ showing the intensity $|{\cal{E}}^\prime_{x,y,z} |^2$ and phase $\varphi^\prime_{x,y,z}$ distributions as visualized in Fig. 3a (simulation). As stated above, by the additional phase vortex in the paraxial field we tailored a difference in phase distributions for transverse and longitudinal focal components as observable in $\varphi _{x,y,z}^\prime$. The fields ${\cal{E}}_{x,y}^\prime$ both include a central phase vortex of charge $\ell = 2$ (or two very close singularities of charge $\ell = 1$). Thus, their superposition ${\cal{E}}_t^\prime = {\cal{E}}_x^\prime + {\cal{E}}_y^\prime$ also reveal a similar phase structures $\varphi _t^\prime$ and a symmetric distribution in intensity, as illustrated in Fig. 3b. Crucial is the contribution of ${\cal{E}}_z$ since its phase differs from the phase of transverse components with only embedding a singular central phase vortex: as a consequence, in the overall superposition ${\cal{E}}^\prime = {\cal{E}}_t^\prime + {\cal{E}}_z^\prime$, we observe a transverse variation between constructive and destructive interference of ${\cal{E}}_t^\prime$ and ${\cal{E}}_z^\prime$. This results in the appearance of an asymmetric distribution for $|{\cal{E}}^\prime |^2$ and, thus, for the fluorescence rate *R*(*x*, *y*), as presented in Fig. 3b. This asymmetric shape is distinctive of the tailored focal fields, only appearing due to the interaction of respective transverse and non-negligible longitudinal components, their amplitude and their relative phases. This interaction is similarly observed for random **d**(*x*, *y*) whereby *d*_*z*_ > 0 is an essential condition provided by our SAMs. Vice versa, the observation of the characteristic asymmetric shape reveals the significant contribution of longitudinal ${\cal{E}}_z$ components, representing the non-paraxial characteristic which is the most difficult to be visualized. Here, by measuring the distinctive fluorescence distribution irradiated by the monolayer, this most of the time invisible property can easily be detected in a single shot without any data post-processing.

### Differentiating non-paraxial from paraxial fields

**Fig. 4:**
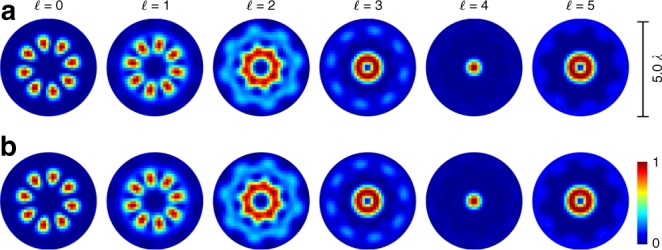
Numerical evaluation of SAM fluorescence for paraxial light fields. SAM (random **d**(*x*, *y*), *d*_*z*_ > 0) is examplarily excited by the far field distribution of (**a**) a vectorial flower (*σ*_12_ = +8) or (**b**) web structure (*σ*_12_ = −8) with additional phase vortices of charge $\ell$. Total normalized intensity is shown

Within the nano-tomographic approach, the observed asymmetry in fluorescence including its intensity (amplitude) and phase distribution mirrors the non-paraxial properties of investigated focal 4D light field. In contrast to non-paraxial fields, paraxial ones only embed negligible longitudinal electric field contributions. As the asymmetric shape is caused by the contribution of longitudinal polarization components, this characteristic shape is not observed in the paraxial regime. Hence, the symmetry of fluorescence represent a fundamental means to differentiate between paraxial and non-paraxial light fields. This can be easily demonstrated by the numerical analysis of fluorescence in the case of applying paraxial fields for the monolayer excitation. For this purpose, we chose the far field distribution of introduced tailored vectorial beams (*σ*_12_ = ±8, $\ell = \{ 0,1,2,3,4,5\}$) to be radiated onto a SAM of random orientation **d**(*x*, *y*), *d*_*z*_ > 0 . The far field distribution is determined according to ${{{\cal{E}}}}_{{\mathrm{far}}} = {\cal{F}}({\mathbf{E}}_{{\mathrm{in}}}(x,{\kern 1pt} y,{\kern 1pt} 0))$ (${\cal{F}}$: Fourier transform, **E**_in_: paraxial input field; see Methods, Eq. (3)). Based on Eq. (1) with ${{{\cal{E}}}} = {{{\cal{E}}}}_{{\mathrm{far}}}$, considering ${\cal{E}}_z \approx 0$, we calculate the respective normalized fluorescence rate distributions, as shown in Fig. 4. Here, Fig. 4a or b belongs to the exciting light field with *σ*_12_ = 8 (flower) or *σ*_12_ = −8 (spider web). Note that, for comparison reasons, we adapted the spatial resolution of shown fluorescence distributions according to the experimental system applied later on (see Methods). Obviously, there are only slight differences visible between results in Fig. 4a, b, even if applied light fields look significantly different in polarization (Fig. 1c, d). Consequently, one cannot distinctly differentiate between a flower or spider web structure by monolayer fluorescence detection. Moreover, all structures are symmetrically distributed (point symmetric), as there is no influence of longitudinal polarization components. In contrast, we expect unique asymmetric fluorescence distributions dependent on *σ*_12_ and $\ell$ to be observed in the case of non-paraxial light fields effected by characteristic ${\cal{E}}_z$ contributions.

### Experimental implementation

Finally, we experimentally prove the functionality of the nano-tomographic SAM-based approach. For this purpose, we investigate the fluorescence distributions excited by tailored non-paraxial 4D light fields to draw conclusions about the fields’ non-paraxial properties. As representative examples, we consider the focal fields (*z* = 0) shown in Fig. 2a, b in experiment and support our results theoretically. Experimentally, fully-structured 4D fields are realized as explained above (Fig. 1a, b), whereby a power of 20 mW is measured at the back-aperture of focusing MO. We place the monolayer probe (random dipole orientation, *d*_*z*_ > 0) in the focal plane of the non-paraxial light field. A high-NA MO (×100, NA = 0.9) in combination with a lens (focal distance: 500 mm) is applied to image the fluorescence in the plane of the monolayer (=focal plane) onto a sensitive CCD camera, whereby excited fluorescence is observed in transmission. The exciting light field (*λ* = 532 nm) is filtered in front of the CCD, so that pure fluorescence (*λ*_fl_ ~594 nm) is detected (details can be found in the Methods).

**Fig. 5:**
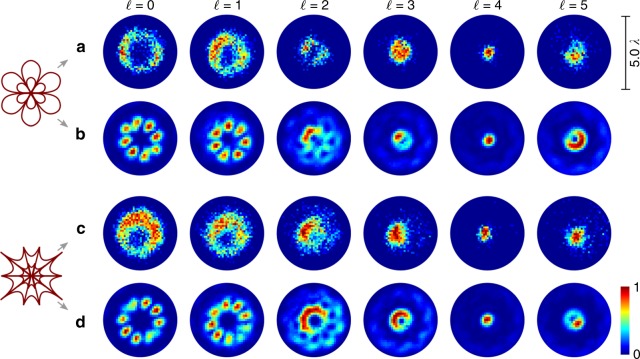
Detecting non-paraxial fields by fluorescent SAM. The normalized fluorescence intensity of tightly focused vectorial flower or web (*σ*_12_ = ±8) with additional phase vortices of charge $\ell$ is analyzed experimentally (**a** and **c**, respectively) as well as numerically (**b** and **d**, respectively), proving the detection of non-paraxial properties. Shown scale corresponds to focal size

**Fig. 6:**
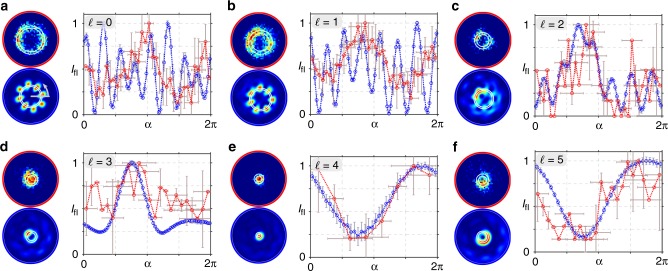
Graphical analysis of fluorescence for focused flower configurations. **a**–**f** *σ*_12_ = 8, $\ell = \{ 0,1, \ldots ,5\}$. Studied intensity images are shown to the left of each graph (experiment: red edged, simulation: blue edged). In graphs, the mean intensity *I*_fl_ in an angular segment of a ring shaped subspace (white marks in intensity images) is shown in dependence of angular position *α* (red: experiment, blue: simulation). Intensity errors are given by standard deviation, experimental angular errors are calculated on the basis of the point spread function of the imaging system

**Fig. 7:**
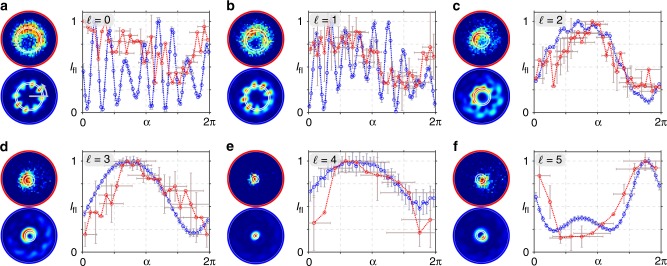
Graphical analysis of fluorescence for focused web configurations. **a**–**f** *σ*_12_ = −8, $\ell = \{ 0,1, \ldots ,5\}$. Studied intensity images are shown to the left of each graph (experiment: red edged, simulation: blue edged). In graphs, the mean intensity *I*_fl_ in an angular segment of a ring shaped subspace (white marks in intensity images) is shown in dependence of angular position *α* (red: experiment, blue: simulation). Intensity errors are given by standard deviation, experimental angular errors are calculated on the basis of the point spread function of the imaging system

In Fig. 5, we present the experimentally measured (a and c) as well as theoretically calculated (b and d) fluorescence intensity (normalized) when the tightly focused vectorial flower and web structure with additional phase vortices $\ell$ are applied as exciting beam. Both, experiment and simulation (spatial resolution adapted to experimental system, see Methods), demonstrate a change in the measured transverse fields diameter with increasing $\ell$, which mirrors numerical calculated focal fields in Fig. 2. Further, simulations show characteristic asymmetric transverse distributions, especially visible for $\ell \ge 2$, which are clearly affirmed by experimental results (deviation explained in Discussion) . Crucially, these detected fluorescence distributions prove the existence and significant contribution of longitudinal polarization components of the non-paraxial field. We confirm this observation by detailed analysis of fluorescence intensity in dependence on angular position (for details see Methods), presented in Figs. 6 and 7. Here, we averaged theoretically calculated (blue edged image) and measured (red edge image) intensity values in angular segments of a ring shaped subspace, marked white. Respective graphs show these mean values as a function of angular position *α* with respective errors (see Methods), additionally proving the agreement of experiment and theory as well as observed asymmetry. Beyond, in contrast to paraxial measurements, the differentiation between flower and web configurations is facilitated, as particularly visible in the graphical analysis (see, e.g., results for $\ell = 2$, Figs. 6 and 7c). Hence, we distinctly evince the dependence on the non-paraxial polarization, amplitude and phase configuration of our nano-tomographic approach. Consequently, our fast single-shot method based on a SAM, not requiring any data post-processing, facilitate the direct qualitative visualization of non-paraxial characteristics, enabling the identification and investigation of focal field properties.

## Discussion

Nowadays, a broad range of different applications is awaiting the inclusion of 4D structured light fields. In particular, the optimal implementation of 4D nano-materials with optically addressable functionality demands an adequate 4D counterpart, namely, precisely tailored 4D light fields. Due to the complexity and nano-scale of 4D non-paraxial fields including its 3D polarization nature, there is a lack of appropriate analysis methods required for the in-depth evaluation and application of these focal fields. Fast, single-shot nano-tomographic techniques are demanded enabling the immediate analysis of focal field properties, whereby this demand is not satisfied by currently proposed methods. These are typically based on slow scanning procedures in combination with the essential precise knowledge of scanning probe characteristics and complex post-measurement algorithms. Here, we presented and theoretically as well as experimentally verified a single-shot nano-tomographic approach without any data post-processing, fully reacting to the current demand and finally enabling the effective implementation of next generation 4D nano-materials.

Considering an inverse approach, we apply the concept of molecular self-assembly to form functionalized 4D nano-surfaces of randomly oriented, polarization sensitive rhodamine B (sulforhodamine B silane), including the key feature of *d*_*z*_ > 0, as spatially resolved nano-detectors for non-paraxial 4D light fields. If being irradiated by 3D polarization structures, we observe spatial fluorescence distributions being strongly related to the exciting non-paraxial electric field including its amplitude, phase as well as polarization. Due to the contribution of longitudinal field components ${\cal{E}}_z$, non-paraxial fields result in characteristic asymmetric fluorescence structures, as we demonstrated in theory and verified experimentally. Thus, the demanded direct and fast identification and investigation of non-paraxial fields is presented.

Deviations of experimental from theoretical results originate from, for example, non-exact perpendicular orientation of the probe in relation to the beam’s optical axis, or, in particular, the limited resolution of the detecting imaging system in combination with low fluorescence power (compared to background noise) and the self-excitation of fluorescent molecules. These reasons are also reflected by error bars in graphs of Figs. 6 and 7. While intensity errors represent the standard deviation of calculated mean values (simulation and experiment), experimental angular positions reveal relatively large error bars determined on the basis of the imaging system’s point spread function (PSF) for a self-luminous point (=SAM molecule) and self-excitation of neighboring molecules (for details see Methods). The respective transverse position of each molecule has an error of approximately ±176 nm (~±0.33*λ*), impeding the resolution of, e.g., the individual intensity spots visible in simulations for $\ell = 0$ or $\ell = 1$ (Figs. 6a, b and 7a, b). Note that the system’s resolution can be increased by decreasing the fluorescence wavelength by choosing another molecule and/or adapting the imaging MO and lens (see Methods), whereby the limited resolution of the sensitive fluorescence CCD camera need to be considered as well. Basic spatial resolution provided by the monolayer is at the nanometer scale, dependent on molecule size. The same applies for resolution in *z*-direction: The nanometer thickness of monolayers only allows a specific plane of the non-paraxial light field to excite molecules. Subsequently, the detection of excited fluorescence follows the typical rules for the longitudinal resolution limit of the microscopic system. Further, fluorescence and, thus, experimental results can be enhanced and self-excitation avoided by applying another fluorescent molecule, which absorbs maximally at the systems wavelength 532 nm and shows low self-excitation, such as quinacridone^42^.

Beyond direct and fast nano-tomographic identification of non-paraxial fields, our approach includes the potential for the full reconstruction of the focal electric field. For this advancement, molecular layers need to be of defined orientation, namely, purely oriented in *x*-, *y*-, and (on average) diagonal direction, i.e., **d** = [1, 0, 0]^*T*^, [0, 1, 0]^*T*^ and $[1,1,1]^T{\mathrm{/}}\sqrt 3$, which could be realized by programmable self-assembly of quinacridone molecules. These sophisticated monolayers will facilitate the spatially resolved single-shot analysis of each electric field component individually by implementing each quinacridone SAM separately: as the field irradiated by molecules is $\propto {\mathbf{d}} \cdot {{{\cal{E}}}}$, transversely oriented SAMs could enable the detection of relative phase and intensity of ${\cal{E}}_{x,y}$. Phases are quatifiable by off-axis interference of fluorescence and a reference beam—of course, in this case the low coherence of fluorescent light, due to the life span of excited molecular states, need to be considered. Subsequently, the diagonally oriented SAM could be used to identify ${\cal{E}}_z$. Note that diagonal orientation is chosen instead of more intuitive *z*-orientation as purely *z*-polarized fluorescence would experience significant transformation in the imaging system impeding the detection of ${\cal{E}}_z$. Beyond, full 4D analysis can be implemented by scanning the three-dimensional volume ${{{\cal{E}}}}(x,y,z)$ in *z*-direction by the monolayers. Hence, different *z*-slices of the non-paraxial field will be investigated, whereby, 4D tomography in nano-scale resolution is facilitated since monolayers of nanometer thickness are applied.

It is worth mentioning that, naturally, our approach cannot only be used for the investigation of non-paraxial light fields but, finally, paves the way for the pending advancement of a broad range of applications awaiting the inclusion of 4D structured fields. For instance, in an inversion of our tomographic approach, if 4D light fields are analyzed and, thus, verified or even fully reconstructed in its non-paraxial characteristics, we can apply focal fields for the evaluation of unknown properties of monolayers or of 4D functional nano-systems as assemblies of phase and polarization sensitive nano-particles. Hence, 4D light fields analyzed by our approach represent a useful nano-technological tool for the effective study and implementation of 4D nano-materials.

## Methods

### Holography-based customization of light

SLMs are well-established, dynamic tools for the on-demand formation of structured light. Therefore, for the realization of tailored vectorial flower or spider web structures including additional phase vortices we combine two holographic modulation techniques by applying a reflective phase-only SLM (Holoeye Pluto, parallel aligned liquid crystal HD display) in split-screen-mode^33,41^, as shown in Fig. 1b. The initial light field (collimated and expanded; wavelength *λ* = 532 nm; continuous wave frequency-doubled Nd:YAG laser, company: Coherent) is of horizontal polarization, as the SLM can only modulate horizontally polarized light in phase. In a first step, we tailor the phase of the light field by encoding the desired phase distribution on one half of the SLM, being passed first. We combine the desired phase structure with an additional blazed grating, so that the modulated field is created in the first diffraction order of the hologram^43^, which can be filtered in the far field. By this, the quality of modulation is enhanced and polarization purified.

To add polarization modulation, the phase structured light field is guided to the SLM a second time, while the first half of the SLM is imaged onto the second half by a 4*f*-system consisting of two lenses (L). Note that in Fourier space, i.e., in the focal plane of the first lens, we filter the first diffraction order of the first SLM half by an aperture (A). The polarization modulation is based on a combination of the polarization selective SLM and two quarter wave plates (QWP_1,2_; multiorder wave plates)^33,36^. First, the orientation of QWP_1_ defines the ratio of horizontal and vertical polarization components reaching the SLM. Next, the SLM introduces a chosen, spatially varying phase shift between horizontal and vertical components, as horizontal light can be modulated, whereas vertical components are reflected unaffectedly. Last, QWP_2_ recombines horizontal and vertical components, dependent on its orientation. In the transverse plane of the modulated light field all states of polarization located on a ring on the Poincaré unit sphere, spanned by normalized Stokes parameters, can be realized. The radius and position of the ring is affected by the QWPs’ orientation. Consider that the modulation of polarization by this method results in an additional phase variation^36,37^, which is corrected by according phase modulation on the first half of the SLM.

For the creation of vector beams, consisting of only linear states of polarization, both wave plates are set to −45° with respect to horizontal input polarization. In this case, the applied hologram is equal to the generated field’s (Stokes field) phase Φ_12_^36,39,40^, defined by the Stokes field *Σ*_12_ = *S*_1_ + i*S*_2_ = *A*_12_ exp(iΦ_12_). Following this, we can directly choose the order of the created vector field or, more precisely, the included V-point singularity index *σ*_12_ by the choice of the hologram.

### Fully-structuring focal 4D fields

After generating the tailored paraxial beam, the phase and polarization structured field can be transformed into the non-paraxial regime. For this purpose, we image the second half of the SLM (Fig. 1a) onto the back-aperture of the applied MO (Fig. 1b). Here, a 2*f*_1_–2*f*_2_system is applied as imaging system adapting the transverse size of the paraxial field to the size of the back-aperture (slightly overfilled back-aperture). In the Fourier plane between lenses, we filter the zeroth diffraction order of the SLM, which includes the modulated paraxial field. Note that guiding mirrors (silver mirror; dichroic mirror, Thorlabs DMLP605R) are chosen to have almost no effect on the polarization. However, deviations of the intended field, investigated at the position of the back-aperture of the focusing MO, are rectified by correction holograms on the SLM^41^. By tightly focusing this corrected, paraxial field by the MO (Nikon, TU Plan ELWD, ×100, NA = 0.8 in air, working distance 4.5 mm), a non-paraxial light field 4D structured in amplitude, phase and polarization, i.e., a fully-structured^32,34^ focal field is formed. The respective degrees of freedom can be customized by the choice of holograms on the SLM.

Within our results we present focal fields created by combining higher-order vector beam, whose polarization structure is of flower- or spider-web-shape, with additional phase vortices (Fig. 2, NA = 0.8, *z* = 0). If a flower (web) of index *σ*_12_ without additional phase modulation ($\ell = 0$) is tightly focused, a focal intensity landscape resembling a |*σ*_12_ − 2|-fold dark star (bright flower) is realized^10^. This means, by the index *σ*_12_ we are able to tailor the total intensity. In addition, the ratio of intensity contributions assigned to transverse and longitudinal polarization states can be varied as indicated by the “max” or “mean” values (ratio of maximum or mean value of $|{\cal{E}}_{x,y,z}|^2$ to the maximum or mean value of $|{{{\cal{E}}}}|^2$, respectively; see ref. ^10^). Another tool for further tailoring the focal field is the addition of phase vortices of chosen charge $\ell$ to the input light field. As presented in Fig. 2, the total focal intensity $|{{{\cal{E}}}}(x,y,0)|^2$ as well as contributions $|{\cal{E}}_{x,y,z}(x,y,0)|^2$ vary depending on $|\ell |$. For example, the transverse size firstly decreases ($|\ell | \le 4$) then increases again ($|\ell | > 4$) with growing $|\ell |$. Moreover, the max and mean values are changing. Interestingly, for the focused flower (a) as well as web (b) structure a Gaussian-like distribution for $|{{{\cal{E}}}}|^2$ is achieved if a vortex of charge $|\ell | = |\sigma _{12}|/2 = 4$ is applied, which we verified by simulations for different *σ*_12_. In this case, the contribution of ${\cal{E}}_z$ is relatively small (see max). Furthermore, distributions for a focused flower (a) with additional vortex $\ell = \pm 3$ (±5) resembles the ones for a focused web (b) with $\ell = \pm 5$ (±3). Here, for $|\ell | = |\sigma _{12}/2 - 1|$ a very strong $|{\cal{E}}_z|^2$ contribution of Gaussian shape is created, whereas for $|\ell | = |\sigma _{12}/2 + 1|$ longitudinal contributions are significantly smaller and of donut shape. Note that, besides tailoring intensity landscapes in the focal plane (*z* = 0), the additional modulation of phase results in the customization of the total focal volume ${{{\cal{E}}}}(x,y,z)$ as indicated in ref. ^44^.

### Analyzing non-paraxial fields

Tailored non-paraxial 4D fields are analyzed by a fluorescent SAM. For this purpose, the probe, consisting of the monolayer produced on a glass cover slip (refractive index *n* = 1.33, thickness 170 μm) is placed in the focal plane of the non-paraxial field. Note that the fluorescent molecules are directly irradiated by the light field, thus, the probe is placed with the monolayer at the bottom on its holder (monolayer-glass-order in beam propagation direction, see Fig. 1a). By this, aberration effects occurring when the beam is transmitted trough the cover slip are avoided, as the MO focuses is aberration free in air. Excited fluorescence is observed in transmission, passing through the cover slip. For collecting scattered fluorescence and imaging the fluorescence in the plane of the monolayer (=focal plane) onto a CCD camera (Photometrics CoolSNAP MYO), we apply a high-NA MO (Nikon C-AB Abbe Condenser, ×100, NA = 0.9) in combination with a lens (focal distance: 500 mm). The exciting light field (*λ* = 532 nm) is filtered in front of the CCD (longpass filter, Thorlabs FEL0550), so that pure fluorescence (*λ*_fl_ ~ 594 nm) is observed . Note that noise within recorded fluorescence images is reduced by taking the mean value of ten images and applying background subtraction.

### Self-assembled monolayers

**Fig. 8:**
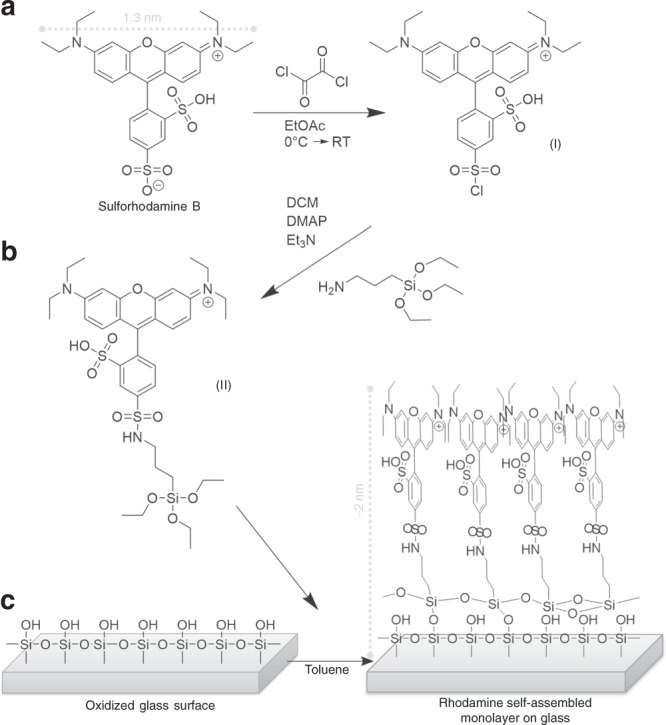
Preparation and synthesis of SAMs of sulforhodamine B silane on glass. The preparation of **a** sulforhodamine B chloride and, subsequently, **b** sulforhodamine B silane, as well as **c** the final formation of the SAM are illustrated (idealized visualization)

For the fluorescent SAM a sulforhodamine B silane (C_36_H_51_N_3_O_9_S_2_Si) was used because of its strong fluorescence and wide application on surfaces as for example in bio arrays^45^. The xanthene substructure in rhodamine is well known and used in a great variety of fluorophores^46,47^. Sulforhodamine B (in solution) has its absorption maximum at a wavelength of 558 nm and its emission maximum at 576 nm, and is therefore a red emitting fluorophore. Further, rhodamine has a rigid structure with a broad *π*-system with a length of about 1.3 nm. Note that depending on the tilting angle of the molecule on the surface its transverse dimension may vary between 1.3 nm and approximately 2 nm (Fig. 8). The preparation and synthesis (see Supplementary Methods) of the SAMs of the sulforhodamine B silane on glass (*λ*_abs_ = 572 nm; *λ*_fl_ = 594 nm) is depicted in Fig. 8 (idealized structures) . The successful formation of the densely packed molecules to a monolayer has been proven by surface analysis (see Supplementary Methods), which shows the expected and desired assembly of triethoxysilanes reacting with glass (SiO_2_) surfaces^27–31^.

The structure of the SAM is dependent on the arising covalent bonds between the silane and the surface and the interactions between the sulforhodamine B moieties in the backbones of the silane. Thus, the sulforhodamine B molecules self-assemble in that sense that they will interact and assemble in a spatially ordered way due to *π*–*π* stacking. The resulting SAM consists of nano-scale domains in which molecules exhibit similar orientations. However, the whole SAM on the glass surface reveal a random orientation on a micrometer level of dipole moments ***d*** with positive *d*_*z*_ due to the defined bonding site of rhodamine B on silane. In addition, aromatic dyes typically tend to stack due to their *π*-system, which may vary the SAM’s characteristics. Note that, due to overlapping of absorption and emission spectra, neighboring rhodamine units can excite each other (self-excitation). We expect a maximum interaction distance of three to four molecules within the monolayer.

SAMs were characterized in detail by different analytic methods. Analytical data can be found in the Supplementary Methods.

### Numerical approaches

To simulate the expected focal field distributions as well as fluorescence images, we applied numerical methods. As described in refs. ^10,48^, we numerically calculate the non-paraxial electric field ${{{\cal{E}}}}(x,y,z) = [{\cal{E}}_x(x,y,z),{\cal{E}}_y(x,y,z),{\cal{E}}_z(x,y,z)]^T$ (here for (*z* = 0)) by solving Richards and Wolfs’ integrals^49^ fast Fourier transform (FFT) operations (NA = 0.8, refractive index *n* = 1). For this purpose, the input (transverse) vectorial light field of order *σ*_12_ with additional phase vortex of charge $\ell$ is described as

3 $${\mathbf{E}}_{{\mathrm{in}}} = \left[ {\cos \left( {\frac{{\sigma _{12}}}{2}\cdot \phi } \right),{\kern 1pt} \sin \left( {\frac{{\sigma _{12}}}{2}\cdot \phi } \right)} \right]^T\cdot \exp \left( {{\mathrm{i}}\ell \phi } \right),$$

whereby *ϕ* represents the polar angle.

For the calculation of the resulting fluorescence, we utilize Eq. (1). Considering the size of fluorescent molecules as well as built islands (see above), we assume *N* × *N* = 1000 × 1000 randomly oriented molecules in the respective numerical field of view (FoV) of the calculated non-paraxial light field ((*x*, *y*)-plane, *b* × *b* with *b* = 256 px). Thus, we create a three-dimensional array **d**(*u*, *v*) = [*d*_*x*_(*u*, *v*), *d*_*y*_(*u*, *v*), *d*_*z*_(*u*, *v*)]^*T*^ with *u*, *v* ∈ [0, *N*] reflecting the number of molecules. Then, we rescale the respective array to the numerical size of the FoV (**d**(*u*, *v*) with *u*, *v* ∈ [0, *N*] → **d**(*x*, *y*) with *x*, *y* ∈ [0, *b*]), so that one FoV pixel embeds *N*/*b* molecules. Next, we calculate the transversely varying fluorescence rate by Eq. (1). In a last step, when we compare simulations and experimental results, we consider the spatial resolution achieved by the system imaging the fluorescent monolayer onto the CCD camera (pixel size: 4.54 μm). Thus, we sum up 5 × 5 adjacent pixels of the numerical FoV so that the spatial resolution is decreased according to the experimental system. Note that the PSF of the imaging system (see below) is not considered here.

### Graphical fluorescence analysis

In order to analyze our experimental in comparison to numerical data in detail, we study the fluorescence intensity dependent on the azimuthal angle. Results are presented in Figs. 6 and 7. For this analysis, we first choose a ring-shaped subspace within fluorescence images, as marked in white in experimental (red edged) and numerical images (blue edged; original resolution). The ring is positioned in such a way that it embeds most conspicuous characteristics of intensity images while having a width of 2–3 pixels in experiment (10–12 pixels in numerics). In the next step, the ring is devided into angular segments of size *δα* with at least including three intensity pixels in the experimental case (experiment or simulation: *δα* = 10° to 45° or *δα* = 5° to 10°). We calculate the mean values of the normalized intensity per angular segment within the ring and plot these in dependence on the respective angular position *α* (central value of angular segment; see Figs. 6 or 7a, bottom left). Experimental and numerical data points are visualized by red and blue circles within graphs, respectively.

For experiment and simulation, the errors in intensity represent the standard deviation of the respective mean value. The angular position error Δ*α* for the experimental results considers the PSF of the imaging system and the self-excitation of neighboring molecules. More precisely, we assume each molecule of the SAM as a self-luminous point in the microscopic imaging system, so that the transverse position error (Cartesian coordinates) of this point can be described by the half of the full width half maximum (FWHM) of the PSF given by^50^

4 $${\mathrm{FWHM}} = \frac{{0.51\lambda _{{\mathrm{fl}}}}}{{{\mathrm{NA}}_{{\mathrm{im}}}}} \approx 337{\kern 1pt} {\mathrm{nm}},$$

(wavelength of fluorescence *λ*_fl_ = 594 nm; NA of imaging MO NA_im_ = 0.9). Further, we consider the possible self-excitation with a maximum interaction distance of four molecules, i.e., an additional error in transverse position of ±4 ⋅ *s*, *s* = 2 nm (*s*: long axis of fluorescent molecule). Hence, in total, we calculate a transverse position error of

5 $$\begin{array}{c}\Delta x = \Delta y = \pm \left( {\frac{{{\mathrm{FWHM}}}}{2} + 8{\kern 1pt} {\mathrm{nm}}} \right)\\ = \pm 176{\kern 1pt} {\mathrm{nm}} \approx \pm 0.33\lambda .\end{array}$$

From this, we can derive the angular error via propagation of uncertainty, resulting in

6 $$\Delta \alpha = \Delta x\cdot (\sin \alpha - \cos \alpha )/r{,}$$

with *r* as the central radius of the ring subspace. Obviously, by increasing NA_im_ or decreasing *λ*_fl_ one can reduce the FWHM and, thereby, Δ*x* or Δ*α*, which improves lateral imaging resolution.

## Supplementary information

Supplementary Information

## Data Availability

Experimental source data are available from the corresponding author upon reasonable request.
