# Supplementary material for: Polarization nano-tomography of tightly focused light landscapes by self-assembled monolayers
Source: Nat Commun. 2019 Sep 20;10:4308. doi: 10.1038/s41467-019-12127-3 (PMC6754390; doi:10.1038/s41467-019-12127-3)
Supplement: Supplementary file 1 — Supplementary Information [file 41467_2019_12127_MOESM1_ESM.pdf]

# Supplementary information: Polarization nano-tomography of tightly focused light landscapes by self-assembled monolayers

Eileen Otte et al.

## SUPPLEMENTARY METHODS

### Synthesis of self-assembled monolayers

The preparation and synthesis of fluorescent self-assembled monolayers (SAMs) is visualized in Fig. 8 of the main article, illustrating an idealized structure of resulting monolayers for small areas (3-10 molecules next to each other; large areas will be functionalized more randomly). The procedure was done as follows.

i. Sulforhodamine B chloride (I), Fig. 8**a**: A solution of sulforhodamine B (4.95 ml, 8.53 mmol, 1.00 eq) in dichlormethane (DCM, 60 ml) was cooled to 0°C. Afterwards oxalyl chloride (3.66 ml, 42.65 mmol, 5.00 eq) was added drop wise. Dimethylformamide (DMF, 100 µL) was added as a catalyst and the reaction was stirred over night. After that the solvent was removed under reduced pressure resulting the product (1) in 96% yield.

ii. Sulforhodamine B silane (II), Fig. 8**b**: To a solution of (I) (500 mg, 0.87 mmol, 1.00 eq) in DCM (5 ml), dimethylaminopyridne (5.30 mg, 0.04 mmol, 0.05 eq) and triethylamine (175 mg, 1.73 mmol, 2.00 eq) were added. The reaction mixture was cooled to 0°C and (3-Aminopropyl)triethoxysilane (APTES; 163 mg, 0.74 mmol, 0.85 eq) was added drop wise. The reaction was allowed to heat up to room temperature and was stirred over night. Subsequently, again the solvent was removed under reduced pressure. The resulting mixture was solved in DMF and extracted with pentane to obtain (II) in 81% yield.

   *Analysis*:

   Calculated mass for [$C_{36}H_{51}N_3NaO_9S_2Si^+$]: 784.2728 m/z.
   Detected mass for [$C_{36}H_{51}N_3NaO_9S_2Si^+$]: 784.2752 m/z (by mass spectroscopy).

   [$^1$H-NMR, 400 MHz, Methanol-d4]: 7.12 (m, 1H), 7.01 (m, 1H), 6.93 (m, 1H), 6.77 (t, $J$ = 2.0 Hz, 2H), 6.68 (dt $J$ = 7.7, 2.0 Hz, 2H), 6.58 (dt, $J$ = 7.7, 2.0 Hz, 2H), 3.80-3.85 (m, 2H), 3.60-3.68 (m, 14H), 3.56 3.51 (m, 2H), 3.32-3.37 (m, 2H), 1.29 (t, 9H), 1.25 (t, 12H).

iii. Preparation of sulforhodamine B SAMs on glass, Fig. 8**c**: Glass surfaces of a size of $1.6 \times 1.6\,cm^2$ were sonicated in pentane, acetone and deionized water for 3 min each. Then they were immersed into a freshly prepared solution of piranha ($H_2SO_4/H_2O_2$ = 3/1) and stirred for 30 min, before they were extensively washed with deionized water and dried under an argon stream. The clean and dry surfaces were then immersed in a freshly prepared solution of sulforhodamine B silane (42 mg, 55 mmol) in toluene (20 ml) for 1 h. Afterwards the surfaces were washed with DCM, ethanol and deionized water.

### Characterization of self-assembled monolayers

The glass surfaces were characterized by several techniques including fluorescence microscopy, atomic force microscopy (AFM), UV/vis spectroscopy, fluorescence spectroscopy and X-ray photoelectron spectroscopy (XPS).

i. XPS measurements demonstrate the successful formation of a SAM of sulforhodamine B silane on the glass substrate. The C 1s spectra (Supplementary Figure 1**a**) shows two specific signals. A smaller signal at 285.0 eV is related to C-C-bonds whereas a broader signal appears at 286.7 eV for C-N- and C-O- and C-S-bonds. In the O 1s spectra (Supplementary Figure 1**b**) also two signals appear, which correspond to Si-O-bonds (530.1 eV) and C-O-as well as S=O bonds (531.4-533.2 eV). The N 1s spectra (Supplementary Figure 1**c**) also indicates a bonding of sulforhodamine B silane to the glass surface. The two identifiable signals can be allocated to the C-N-bonds (399.8 eV) and S-N-bond (397.7 eV). The S 2p spectra (Supplementary Figure 1**d**) shows a signal for the sulfate groups (168.7 eV) and a Si-signal (154.1 eV).

ii. A further proof of a successful reaction was given by UV/vis- and fluorescence spectroscopy. In Supplementary Figure 2 the measured absorption and emission spectra can be seen. A maximum absorption for the suforhodamine B silane functionalized surface was detected at a wavelength of 572 nm. The maximum emission was detected at a wavelength of 594 nm. Both maxima are in a good agreement with sulforhodamine B spectra form literature.

iii. To visualize the monolayer of sulforhodamine B silane is smooth and without larger aggregates a fluorescence microscopy image was taken (Supplementary Figure 3). As seen in that image a homogeneous and clean surface was detected (equivalently expected for smaller scales). Defect parts with a higher or lower emission due to aggregation were not found.

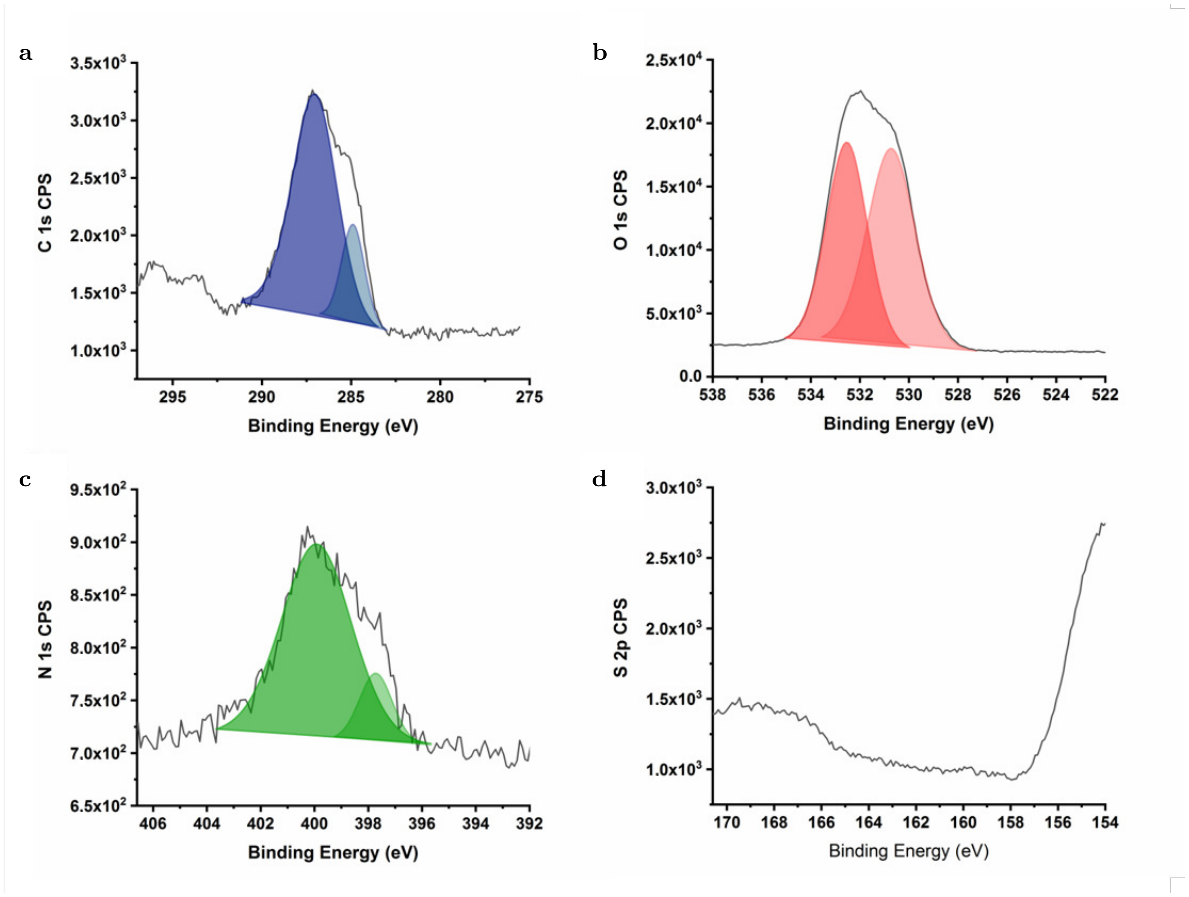


Supplementary Figure 1. Elementary analysis by X-ray photoelectron spectroscopy of sulforhodamine B silane SAM on glass surface. (**a**) C 1s, (**b**) O 1s, (**c**) N 1s, and (**d**) S 2p spectrum.

iv. The results shown above are further confirmed by AFM. If aggregation is present on the sulforhodamin B silane functionalized surfaces, it should be visible in the AFM image due to the size of the molecules. Instead a flat and homogeneous surface is detected (Supplementary Figure 4**a**) which is very similar to a surface of a unfunctionalized glass surface (Supplementary Figure 4**b**) and leads to the assumption of a well defined monolayer of the sulforhodamine B silane.

## Chemicals and instruments

All chemicals were purchased from Sigma-Aldrich Chemie GmbH, Taufkirchen, abcr GmbH & Co. KG, Karlsruhe or TCI Deutschland GmbH, Eschborn and were used without further purification.

UV/vis spectra were obtained on a double beam spectrometer JASCO V650 (JASCO Labor und Datentechnik GmbH, GrossUmstadt) and a JASCO V750 (JASCO Labor und Datentechnik GmbH, GrossUmstadt) at 25°C. For measuring on surface, glass slides were directly placed in the measuring chamber. The spectrometer was operated using Spectra Manager 2 version 2.14.06 (JASCO Labor und Datentechnik GmbH, GrossUmstadt). For glass surfaces a reference slide was placed in the reference chamber.

AFM imaging was performed using a NanoWizard 3 from JPK Instruments. For tapping mode Veeco RTESPW tips and Nanosensors PPP-NCHAuD-50 tips were used. The AFM was typically operated with a set pint between 0.8 and 0.9 V. The line rate was set between 0.8 and 1.5 Hz with an image resolution of $512 \times 512$ pixels. Resulting data were analyzed with Gwyddion 2.52.

X-ray photoelectron spectroscopy measurements were performed with an Axis Ultra DLD (Kratos Analytical Ltd, UK). A monochromatic Al K$\alpha$ source ($h\nu = 1486.6$ eV) at 10 mA filament current and 12 kV filament voltage source

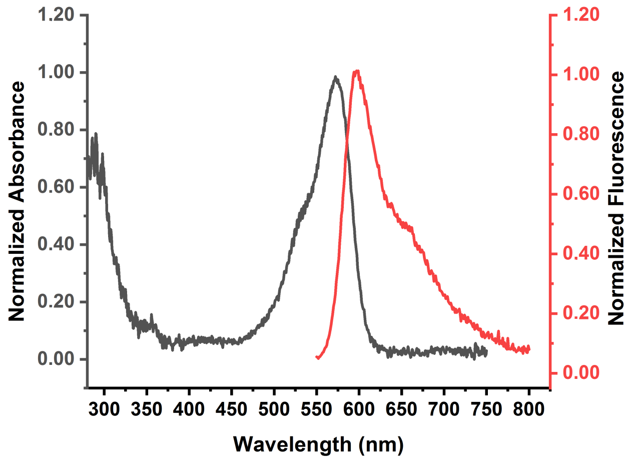


Supplementary Figure 2. Absorption and emission spectra of a sulforhodamine B silane SAM on a glass surface measured by UV/vis and fluorescence spectroscopy.

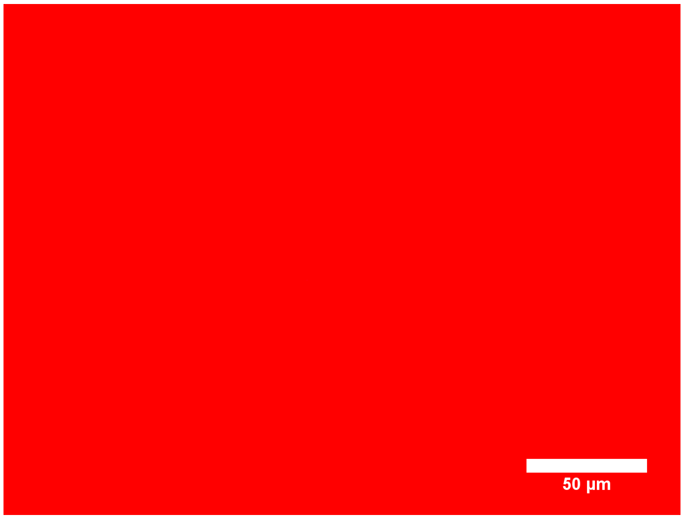


Supplementary Figure 3. Fluorescence microscopic image of a sulforhodamine B silane SAM on a glass surface. No defects could be detected (50× magnification).

energies was used. The pass energy was set to 20 eV for high resolution and to 160 eV for survey scans. A charge neutralizer was used to compensate for sample charging. All measurements were carried out in 'hybrid mode'. The number of sweeps was depending on the element and set between 5 to 15. The X-ray beam had the dimension of 300 µm × 700 µm. The data were evaluated with CasaXPS (version 2.3.15, Casa Software Ltd, UK) and the spectra were calibrated to aliphatic carbon (C1s = 285 eV).

Fluorescence microscopy was performed with an Olympus BX 53 microscope with a top viewing Olympus XC 10 camera. As light source for irradiation a X-Cite® Series 1200 from Lumen Dynamics was used. For fluorescence images the following interference filters were used: UV-irradiation: ET-DAPI, U-F49000. For irradiation from 450-500 nm (blue light): U-FBW. For irradiation from 525-560 nm (green light): U-FGW. Images were taken and processed with OLYMPUS Stream Start 1.8, further processing was done with ImageJ 1.48v.

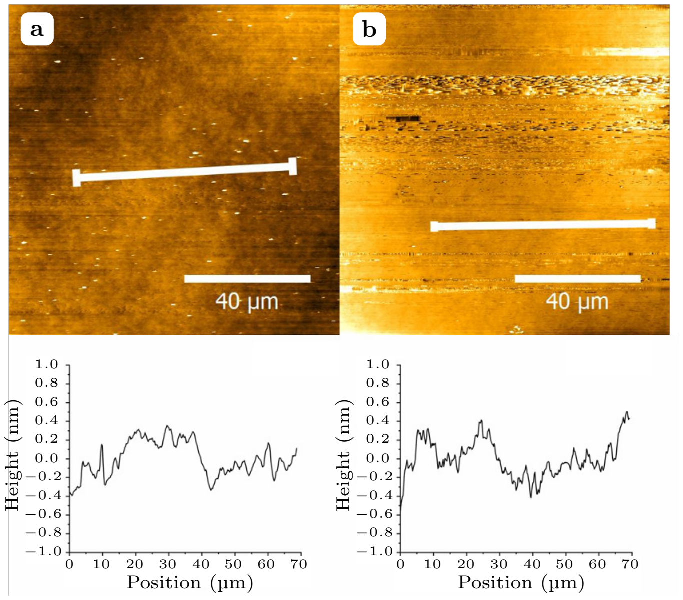


Supplementary Figure 4. Atomic force microscopic images with corresponding height profile below. (**a**) sulforhodamine B silane SAM on a glass surface and (**b**) a clean glass surface.
